# Supplementary material for: Screen Your Way Study Protocol: Embedding community driven models to increase cervical screening via HPV self-collection to improve cervical cancer outcomes for Aboriginal and Torres Strait Islander people
Source: PLoS One. 2026 Apr 22;21(4):e0336004. doi: 10.1371/journal.pone.0336004 (PMC13102237; doi:10.1371/journal.pone.0336004)
Supplement: S2 File — (DOCX) [file pone.0336004.s002.docx]

Inclusivity in global research

PLOS’ policy on inclusivity in global research aims to improve transparency in the reporting of research performed outside of researchers’ own country or community and ensures that PLOS publications reporting global research adhere to high standards for research ethics and authorship. Authors of relevant research articles may be asked to complete the questionnaire below, which outlines ethical, cultural, and scientific considerations specific to inclusivity in global research. This questionnaire may be requested when researchers have travelled to a different country to conduct research, if research uses samples collected in another country, research with Indigenous populations or their lands, or if research is on cultural artefacts. Researchers travelling to another country solely to use laboratory equipment will not normally be required to complete the questionnaire. However, the questionnaire can be requested at the journal’s discretion for any submission – if you have been requested to complete this questionnaire by the PLOS journal you submitted to, please do so.

Please complete the questionnaire below and include this as a Supporting Information file with your manuscript. Note that if your paper is accepted for publication, this checklist will be published with your article in the supporting information files. Please ensure that you reference the checklist in the main body of your manuscript. We suggest adding a subsection ‘Inclusivity in global research’ to your Methods section and adding the following sentence: “Additional information regarding the ethical, cultural, and scientific considerations specific to inclusivity in global research is included in the Supporting Information (SX Checklist)”

The questions have been designed to be applicable to a wide range of study types, and there are subsections for both human subjects research and non-human subjects research. If any of the questions are not relevant to your research please mark them as “N/A” as appropriate.

**Ethical considerations, permits and authorship**

*This section is applicable to all research types.*

Provide details as to who granted permissions and/or consent for the study to take place in the Methods section of your manuscript. This should include the names of **all** ethics boards, governmental organizations, community leaders or other bodies that provided approval for the study. If individuals provided approval refer to these people by their role or title but do not list their name(s).

Reported on page number: Ethics approvals are provided on line 189. Approval has been obtained from the AIATSIS Research Ethics Committee (REC-0092), the Aboriginal Health and Medical Research Council of New South Wales Ethics Committee (2078/23), the Australian National University Human Research Ethics Committee (H/2023/1103), the HREC of the Northern Territory Department of Health and Menzies School of Health Research (HREC 2023-4557), and the Metro South Human Research Ethics Committee (HREC/2025/QMS/115155).

If there were any deviations from the study protocol after approval was obtained please provide details of these changes in the Methods section of your manuscript.
Did this study involve local collaborators that are residents of the country where the research was conducted or members of the community studied? If you do not have any authors from said communities, please provide an explanation for this below.

Reported on page number: There hae been no deviations from the approved study protocol.

This research was open to Aborginal Community Controlled Health Organisations within Queensland, New South Wales and the Northern Territory of Australia. We have consulted with ACCHO peaks within these Jurisdictions. Our positionality (detailed on line 183) and includes Aboriginal and Torres Strait Islander researchers who guide the research. Our Governance processes are outlined on line 282 and includes Aboriginal and Torres Strait Islander Reference Group, Thiitu Tharrmay, at the Australian National University.

Everyone listed as an author should meet PLOS’ criteria for authorship and all individuals who meet these criteria should be included in the author byline, rather than the acknowledgements. For further information please see the journal’s Authorship Policy.

We confirm all authors meet the requirement as per the Authorship Policy

**Human subjects research (e.g. health research, medical research, cross-cultural psychology)**

Did you obtain written informed consent from a representative of the local community or region before the research took place? How did you establish who speaks for the community? Details of written informed consent obtained from study participants should be reported separately in the Methods section of your manuscript.

This research was open to Aborginal Community Controlled Health Organisations within Queensland, New South Wales and the Northern Territory of Australia. We have consulted with ACCHO peaks within these Jurisdictions. Our positionality (detailed on line 209) and includes Aboriginal and Torres Strait Islander researchers who guide the research. Our Governance processes are outlined (line 282) which includes Aboriginal and Torres Strait Islander Reference Group, Thiitu Tharrmay, at the Australian National University.

Our consent processes for recruiting ACCHOs is outlined on line 399 and includes adherence to any local additional consultation or board apporvals to meet local requirements.

How did members of the local community provide input on the aims of the research investigation, its methodology, and its anticipated outcome(s)?

Screen Your Way was designed with input from Aboriginal and Torres Strait Islander peoples working within the ACCHO sector ( SE, KF, CT, MW). The research also includes a dedicated period of co-design to tailor the implementation to each ACCHO, including strategies, methods for implementation and outcome measures to ensure the study meets local community and service needs.

When engaging with the local community, how did you ensure that the informed consent documents and other materials could be understood by local stakeholders?

Services opted into the process through an expression of interest. Informed consent is obtained from a service level representative familiar with research (i.e. CEO, Board) following discussions with the research team who can take the time to explain the research process clearly. Consent can also be tailored to meet local needs.

For Yarns, a plain langage statement is available and will be verbally explained to each particiant to ensure informed consent. This can be facilitated via local service representatives or the research team depending on local preference.

Will the findings of the research be made available in an understandable format to stakeholders in the community where the study was conducted (e.g. via a presentation, summary report, copies of publications, etc.)? Please provide details of how this will be achieved.

Each participating service will receive tailored reports to directly translate findings to the local context.

Dissemination of research findings is outlined on line 652. This will include the creation of reseources beyond academic publications, including translational workshops, evidence briefs and presentations, reports and the creation of resources.

**Non-human subjects research using specimens/ animals collected as part of the study, or those housed in archival collections. Examples include archaeology, paleontology, botany and zoology.**

**N/A**

Did the permission you obtained from a local authority to perform the study include an agreement on access to outputs and benefit sharing? This may include procedures to enable fair distribution of the benefits and resources arising from the research performed. Please include any details of Prior Informed Consent and Benefit Sharing Agreements obtained. These may be required by field-specific regulations, for example the Convention on Biological Diversity (CBD) and the associated Nagoya Protocol.

If the material used in your study was imported, please A) provide the year it was imported and B) indicate whether permits were obtained to import/export the materials used, C) provide details of any permits obtained. If this information is not available, please indicate this.

If you used archival specimens, please state how the material used in your study was acquired by the institute it is held in and provide details of any permits obtained for the original excavations/ sample collection. If this information is not available, please indicate this.

How was the potential cultural significance of the materials collected in your study to local communities considered in your research design? Were Indigenous peoples and/or local researchers and institutions involved with archaeological excavations / collection of specimens? If so, please provide a description of their involvement.

If your manuscript includes photographs of human remains please indicate whether authors obtained permission from descendants or affiliated cultural communities to do so.
